# Society Meetings

**Published:** 1918-03

**Authors:** 


### SOCIETY MEETINGS

Brief reports and announcements of meetings of societies of Western New York. and those of general scope, are requested from Secretaries. Copy should be on hand the fifteenth of the month. Full transactions will be published at cost of composition.

---

**Elmira Academy of Medicine,** February 5th. Program: Dr. Arthur Booth, Blood Transfusion; Dr. Ross G. Loop and Dr. N. H. Soble, subjects not announced.

---

**Rochester Academy of Medicine,** Meeting Feb. 13, speaker from the department of the Surgeon General U. S. Army.

**Erie County Medical Society.** Meeting Feb. 18. Program: Control of Venereal Diseases, Dr. E. H. Marsh, New York; Treatment and Diagnosis of Venereal Diseases, Dr. Grover Wende, Buffalo.

---

The annual meeting of the **Gross Medical Club** was held at the Hotel Statler, Friday, Feb. 15th, Dr. Jas. E. King, Pres. presiding.

Dr. Byron E. Smith of Angola presented a most interesting paper, "The uric acid bugaboo."

He went most carefully into the formation of uric acid in the system and its elimination by the urine; also as to the form, acid urates and uratesm in which it is found. Great stress was laid on the assertion that uric acid, as a causative agent in many of the ills attributed to it, and especially painful conditions, as rheumatism, neuritis, neuralgia, etc., was a sad error, a delusion that should be corrected. Scientifically, but clear and practical, Dr. Smith showed how easily an approximately accurate estimation of uric acid in excess could be detected from the urine. When the urine cools; becomes cider like in appearance, and becomes clear on heating, urates is the cause. In the Heller test for albumin, if a whiteish zone appears in the free urine, this is acid urates; in both conditions they show an excess of uric acid in the system.

In corroboration of his contentions he reported several cases that had been treated weeks for neuritis, rheumatism and like conditions where pain was the important symptom; in all an excess of uric acid was eliminated, recovery taking place very promptly under appropriate treatment as was indicated by an estimation of the alkaline phosphates in the urine by aid of the phosphatometer; the conditions were true nerve involvement as the instrument showed.

Dr. Tyler, Alden. One of the best papers that I have ever listened to. Fully 50% of cases sent to the Sanitarium, diagnosed as rheumatism, and due to uric acid in excess, we find not to be rheumatism, but pain due to some nerve condition. We use the phosphatometer in nearly every case; in a goodly number a low index is found and the phosphorus mixture of Dowd is ordered; most brilliant results has followed.

Dr. Phelps, E. Aurora. Too much cannot be said in praise of this most interesting paper; it certainly has taught me many things that I did not know.

Dr. Geo. F. Cott. I have used the Dowd Phosphatometer for several years; it may not be of much value to the nose and throat specialist, but it is of undoubted great service to the general practitioner; I know from personal knowledge it is used extensively and hear most glowing reports.

Dr. W. H. Baker, Williamsville, I believe Dr. Smith is correct, uric acid as a cause of disease has been an over worked condition.

Dr. Geo. F. Cott, Buffalo, reported a case of cancer of the larynx in which he removed the whole organ some five months ago. The patient at the present time is working every day and feels fairly well.

The election of officers resulted as follows: Dr. Byron E. Smith, Angola, N. Y., President; Dr. Harry R. Trick, Buffalo, N. Y., Vice-President; Dr. Chester C. Cott, Buffalo, N. Y., Secretary. The annual banquet followed the meeting.

